# Validity and reliability testing of the Spanish version of the BESTest and mini-BESTest in healthy community-dwelling elderly

**DOI:** 10.1186/s12877-020-01724-3

**Published:** 2020-11-04

**Authors:** Pilar Dominguez-Olivan, Angel Gasch-Gallen, Esmeralda Aguas-Garcia, Ana Bengoetxea

**Affiliations:** 1 IIS Aragón, Facultad de Ciencias de la Salud, Universidad de Zaragoza, Zaragoza, Spain; 2 Hospital Universitario Miguel Servet, Zaragoza, Spain; 3 Unité de Recherche en Sciences de l’Ostéopathie (URSO). Faculté des Sciences de la Motricité, Université Libre de Bruxelles (ULB), Brussels, Belgium; 4 Instituto de Investigación Sanitaria Biocruces Bizkaia, Barakaldo, Spain

**Keywords:** BESTest, MiniBESTest, Spanish, Balance, Elderly, Reliability, Validity

## Abstract

**Background:**

The Balance Evaluation Systems Test (BESTest) and its abbreviated version, the Mini-BESTest are clinical examination of balance impairment, but its psychometric properties have not yet been tested in European Spanish. We aimed to assess the psychometric properties of BESTest and Mini-BESTest in Spanish in community-dwelling elderly people.

**Methods:**

We designed a cross-sectional transcultural adaptation and validation study.

Convenience sample of thirty (N-30) adults aged 65 to 89 years old without balance problems were recruited. Two physiotherapists assessed participants at the same time. Internal consistency of Spanish BESTest and Mini-BESTest was carried out by obtaining the Cronbach Alpha. The reproducibility between raters was studied with the Intraclass Correlation Coefficient. The Pearson correlation coefficient was calculated by comparing the relationship between the BESTest, mini-BESTest, Berg Balance Scale (BBS) and Falls Efficacy Scale-International (FES-I).

**Results:**

BESTest and Mini-BESTest showed good internal consistency. BESTest and Mini-BESTest total scores showed an excellent inter-rater agreement. There was a significant correlation between total score of the BESTest and the Mini-BESTest (r = 0.65; *p* < 0.001). BESTest had a moderate association with BBS and a strong association with FES-I. Mini-BESTest had a fair correlation with BBS and FES-I. Total scores obtained by women at BESTest and at Mini-BESTest were significantly lower than those reached by men. The differences observed in all the test when disaggregating data by sex require further research.

**Conclusions:**

Spanish versions of BESTest and Mini-BESTest are comprehensible for new raters. They are reliable tools to provide information on which particular balance systems show impairment in community dwelling older adults. Elderly women had a worse quality of balance and a greater perception of their risk of falling.

**Trial registration:**

This study was registered in ClinicalTrials.gov with NCT 03403218 on 2018/01/17.

## Background

Motor control is defined as the ability to regulate or direct the mechanisms essential for balance and movement [1]. It is very complex and involves many different underlying systems. A system must be understood as a set of elements whose parts or components are related to at least some of the other components.

During ageing, motion initiation, postural control and movement are usually impaired by factors involving effectors, sensory and cognitive systems. Specifically for the vestibular system, above 70 years old, a decrease in the number of sensory vestibular cells and a reduction in vestibular nerve fibres and neurons in the vestibular nuclei has been described [2]. Visual function is also impaired, including reduction in visual acuity, depth perception and peripheral vision [3]. In the musculoskeletal system, osteoporosis, sarcopenia and reduction of body muscle strength are established effects to a greater or lesser extent. As regards the effects of ageing in cognition, selective attention is reported to be less effective in the elderly compared to young subjects. Among other degradations, an increase in the attentional cost of posture control and displacement has also been mentioned [4].

Falls are a geriatric syndrome derived from this deterioration and constitute one of the most frequent and potentially significant public health problems [5]. Although there is no clear indication of the factors that imply worse balance for men and women older than 65 years, there appears to be general agreement in the literature that old women have a worse balance than old men [6–8].

The potential of clinical measurements of balance is usually limited to identifying the risk of falling, within the elderly population. The Berg Balance Scale (BBS) is a widely used clinical scale for functional balance and is currently considered as a reference for assessing balance in people with risk of falling due to stroke or Parkinson’s disease [9, 10].

Considered the gold standard, the BBS has been strongly evaluated as valid and reliable but there are still several factors that indicate that the BBS should be used in conjunction with other balance measures. There are few tasks in the BBS to test dynamic balance, which may limit its power to identify sensitive older adults from those who live independently in the community [10]. Also, ceiling effect and floor effect has been reported for the BBS when used with community dwelling older adults [7, 11]. The Falls Efficacy Scale- International (FES-I) is a measure of “fear of falling” or, more properly, “concerns about falling”, which is also suitable for research and clinical practice. Stroke rehabilitation or Spanish studies about sarcopenic obesity or balance in the older people with instability include this instrument [12–14]. The identification of valid, reliable and clinically relevant outcome measures to document and evaluate the evolution or recovery of a health condition is vital in determining the effectiveness of interventions. The Balance Evaluation Systems Test (BESTest) and its abbreviated version (Mini-BESTest) are clinical balance tools that allow the identification of not only the risk of falling, but also which impairments are involved in dysfunctional balance. Having detected the degraded components underlying balance control, specific types of therapeutic intervention for different types of balance problems can be applied [10].

The BESTest has shown to have high correlation with functional gait and balance performance and moderate correlation with self-assessed fear of falling (FES-I) in English, its source language [15, 16].

However, clinical systems used all over the world must not only be translated, but must also be adapted culturally to maintain content validity at a conceptual level across different cultures [17]. Psychometric properties must also be preserved in the destination language. This makes it necessary to analyse its validity before dissemination [18].

The purpose of this study was to translate and adapt BESTest and Mini-BESTest to Spanish (from Spain, Europe) and investigate its validity in elderly healthy subjects without apparent difficulties in balance and locomotion. Secondly, we aimed to study with both instruments the effect of sex-gender in balance.

We hypothesized that BESTest and Mini-Bestest would highly correlate with the Berg Balance Scale (BBS) and would have a low correlation with self-assessed fear of falling in healthy older adults measured by means of Falls Efficacy Scale-International (FES-I).

## Methods

### Study design

A cross-sectional transcultural adaptation and validation study design was used.

### Participants

Participants were 30 community-dwelling elderly subjects (14 males and 16 females) (mean age: 73.3, range: 65–89) recruited with a convenience sampling. Inclusion criteria were: (1) no present health problems relating to balance disturbances; (2) no history of falling in the last three months; (3) the ability to walk 6 m without orthopaedic devices or the assistance from another person; (4) cognitively able to receive three verbal instructions; (5) capable to perform tests without excessive fatigue.

The study followed the Declaration of Helsinki and all the participants received oral and written information about the study and signed the informed consent. Ethical approval was obtained from the Comité Etico de Investigación de Aragón - Spain (CEICA).

### Clinical scales

The BESTest is a 27-item test organized in six sections, using a model of motor control as a theoretical framework [1]. Each item is scored from 3 (normal) to 0 (severe, not capable) points based on time or performance criteria resulting in a total possible score of 108 points, which are converted to a percentage score. Higher scores indicate better balance.

Items are grouped into 6 systems called “Biomechanical Constraints”, “Stability Limits/Verticality”, “Anticipatory Postural Adjustments”, “Postural Responses”, “Sensory Orientation” and “Stability in Gait”.

The mini-BESTest was developed using factor analysis to identify the items of the BESTest that represented dynamic balance, eliminating redundant and insensitive items from the BESTest [5]. It includes tasks from the BESTest sections “Anticipatory Postural Adjustments”, “Postural Responses”, “Sensory Orientation” and “Stability in Gait”, and comprises 14 items scored from 2 (normal) to 0 (severe, not capable), resulting in a total possible score of 28 points, where higher scores indicate better balance.

The BBS consists of 14 functional balance items that focus on static and dynamic balance abilities. Each item is scored from 4 (normal function) to 0 (impossible). The total possible score is 56 points. The interpretation of the result is: ≤20 - wheelchair user, > 20 ≤ 40 - walking with assistance, > 40 ≤ 56 – independent. A score lower than 46 points indicates that the person has a high risk of falling [10].

The FES-I system measures the level of confidence shown by the subject in carrying out basic activities of daily life. It comprises 16 items with a Likert scale of 4. The total possible score is 64 points. Higher scores indicate worse balance [19].

### Transcultural translation process

For the translation of BESTest, Mini-BESTest and their respective instructions, the Beaton recommendations were followed [17]. Firstly, two qualified people translated both instruments from English to Spanish. Then a certified translator back translated the tests to English. The original author of the tests, Fay Horak, reviewed and commented both English documents. All corrections were translated to Spanish and integrated in the managed balance tests. The International System of Units was adopted in the Spanish version of both tests, converting pounds into kilograms and feet and inches into centimetres. Results were rounded off to the nearest unit. A group of physiotherapists instructed in BESTest and Mini-BESTest assessment reviewed the final versions and followed a pre-test study at the Hospital Universitario Miguel Servet (Zaragoza, Spain) with neurological patients and 12 community-dwelling elderly healthy subjects. This training period allowed adjustment of a minor number of instructions to improve items understanding in the cultural context.

### Validation procedure

Feasibility analysis was not necessary because these measurement systems are already applied and validated in other languages [20, 21].

Two trained senior physiotherapists assessed the 30 subjects by means of the BESTest and Mini-BESTest, the BBS and FES-I. Redundant items of BBS and BESTest were assessed together. The Mini-BESTest scores were rated simultaneously with the BESTest scores, so that the corresponding test was only applied once for each subject.

Evaluations were made at the home of each subject, where required clinical equipment was taken. The full session lasted approximately 45 min. The BERG scale was always administered before BESTest; FES-I was fulfilled at the beginning or at the end of the assessment. To avoid fatigue of participants, both physiotherapists scored each item at the same time without communication between them.

The data capture period of the study lasted two months.

### Data analysis

Descriptive statistics were first calculated for age, height, weight and each of the measures with 95% confidence interval (CI) when applicable. Cronbach Alpha was calculated to assess internal consistency of BESTest and mini-BESTest. Values higher than 0.70 were considered to indicate internal consistency of scales. Reliability was calculated via Intraclass correlation coefficient (ICC) and 95% CI using analysis of variance models. Consistent with previous work, an ICC was defined as (1) very good or excellent > 0.75; (2) good, 0.74 to 0.60; (3) fair, 0.59 to 0.40; and (4) poor, < 0.4 [22, 23] . Minimum detectable change (MDC) was calculated starting from the standard error of the measurement and taking into account the standard deviation of the values of all the participants. A 95% CI was applied to calculate the formula.

The Pearson correlation coefficient was calculated to examine criteria validity by comparing the relationship between the BESTest, mini-BESTest, BBS and FES-I. A correlation (r value) between 0 and 0.25 was interpreted as little or no relationship, between 0.25 and 0.5 as a fair relationship, between 0.5 0.75 as moderate, and above 0.75 as a very good to excellent relationship.

The presence of floor and ceiling effects was defined as 15% or more of the participants having the lowest or respectively the highest possible score on the BESTest and the Mini-BESTest [24].

All analyses were conducted with SPSS software, version 22.0 (IBM Statistic for Windows. Armonk, NY: IBM Corp.). The effect of sex-gender was analysed after verifying the variables normal distribution using one-way ANOVA. TIBCO® Data Science – Statistica® was used for this last analysis.

## Results

30 people participated in the study, 14 were men (46.7%) and 16 women (53.3%). They were aged 65 to 89 years old. None of the participants used walking aids. Our cohort do not present anthropometric differences depending on gender (Table 1).

**Table 1:** Characteristics of the study population and measures of the BBS and FES-I central tendency

Measure (units)	Total cohort (*n* = 30)		Men (*n* = 14)		Women (*n* = 16)		F [2, 25] and *p* values
	Mean (SD)	Min-max (CI 95%)	Mean (SD)	Min-Max (CI 95%)^*^	Mean (SD)	Min-Max (CI 95%)^†^	
Age (y)	73 (6.2)	65–89 (70.7–75.3)	72.71 (7.12)	65–89 (68–78.6)	73.25 (5.52)	65–83 (70.3–76.2)	0.05 (0.82)
Weight (Kg)	69 (13.3)	49–92 (64–74)	77.43 (10.40)	62–92 (68.3–81.5)	61.63 (11.11)	49–86 (55.7–67.5)	16.02 (0.00)*
Height (m)	1.62 (0.09)	150–181 (159.1–165.6)	1.68 (0.08)	155–181 (162.3–172.9)	1.57 (0.05)	150–170 (154.1–160)	16.02 (0.00)*
BMI (Kg/m^2^)	26 (3.74)	21–35 (24.7–27.5)	27.21 (3.26)	22–35 (24.7–28.2)	25.06 (3.99)	21–34 (22.9–27.2)	2.57 (0.12)
BBS	50.1 (2.9)	43–52 (48.9–51.2)	51.3 (1.4)	48–52 (50.6–52.3)	49.1 (3.4)	43–52 (47.2–50.9)	5.07 (0.03)*
FES-I	18.7 (1.9)	17–26 (17.9–19.5)	18.1 (1.5)	17–22 (17.1–19.1)	19.1 (2.2)	17–26 (18–20.3)	1.92 (0.18)

^*^ Minimum and maximum with 95% confidence interval. * p < 0.05

Table 1 presents the BBS and FES-I scores as the parameters that illustrate the balance status of our cohort and their own perception about risk of falling. BBS showed significant differences between men (51.3 (1.4)) and women (49.10 (3.40)), (F(1.28) = 5.07, *p* < 0.03, Table 1). Only 3 people (all woman) showed a BBS score under 46, meaning that our cohort had a high risk of falling, although they had not reported falls in the last three months.

None of the participants got the lowest or highest possible score at the BESTest and Mini-BESTest, so no floor or ceiling effects were detected.

The total score (%) for BESTest obtained by the second rater presented a significantly higher score for men than for women (F(2. 27) = 5.28, *p* = 0.01, Table 2).

**Table 2:** Scores obtained by sections and total score of BESTest and Mini-BESTest. Values obtained by each raters

	Rater 1						Post-hoc Scheffé	Rater 2						Post-hoc Scheffé	F [2, 25] & (p) values
	Total cohort		Men		Women			Total cohort		Men		Women			
	Mean	SD	Mean	SD	Mean	SD		Mean	SD	Mean	SD	Mean	SD		
I. Biomechanical Constraints	9.97	2.47	11.00	2.45	9.06	2.17	(0.03)*	10.20	2.20	11.21	2.04	9.31	1.99	(0.01)*	3.22 (0.06)
II. Stability limits/Verticality	18.57	1.85	19.14	1.35	18.06	2.11	NS	18.43	1.94	18.71	1.81	18.19	2.10	NS	1.82 (0.18)
III. Anticipatory Postural Adjustments	15.73	1.18	16.07	1.73	15.44	1.90	NS	15.70	1.72	16.07	1.68	15.38	1.75	NS	0.68 (0.52)
IV. Postural Responses	14.93	1.82	15.79	1.31	14.19	1.90	(0.01)*	14.03	1.92	15.0	1.75	13.19	1.68	(0.01)*	4.48 (0.02)*
V. Sensory Orientation	13.63	1.35	13.71	1.64	13.56	1.10	NS	13.63	1.35	13.64	1.65	13.63	1.10	NS	0.24 (0.78)
VI. Stability in Gait	18.07	1.82	18.71	1.77	17.50	1.71	(0.02)*	18.47	1.78	19.29	1.27	17.75	1.88	(0.07)	3.47 (0.05)*
BESTest total score 0–108	90.90	6.80	94.43	6.03	87.81	6.01		90.47	6.21	93.93	5.91	87.44	4.83		
BESTest (0–108)/108*100	84.17	6.30	84.59	6.49	83.80	6.32	(0.74)	83.77	5.75	86.97	5.48	80.96	4.47	(0.01)*	5.28 (0.01)*
I. Anticipatory postural adjustments	6.57	1.16	6.78	1.19	6.37	1.15	NS	6.53	1.22	6.79	1.25	6.31	1.19	NS	0.54 (0.59)
II. Postural responses	7.03	0.96	7.28	0.99	6.81	0.91	NS	6.50	0.82	6.71	0.82	6.31	0.79	NS	1.08 (0.35)
III. Sensory orientation	5.03	0.96	5.21	1.05	4.87	0.88	NS	5.03	1.03	5.07	1.14	5.00	0.97	NS	0.72 (0.50)
IV. Stability in gait	8.13	1.01	8.57	0.94	7.75	0.93	NS	8.20	1.41	8.57	1.28	7.87	1.09	NS	2.86 (0.07)
Mini-BESTest	21.1	1.6	21.93	1.38	20.37	1.45	(0.01)*	20.8	2.0	21.36	2.50	20.31	1.45	(0.17)	4.5 (0.02)*

*NS* not significant. * p < 0.05

There was a significant difference between groups (women and men) (F(2. 27) = 4.50, *p* = 0.02, Table 2) but only for the first rater (*p* < 0.01).

When analysing the BESTest section scores disaggregated by gender, scores obtained by women were lower than those reached by men, regardless of the BESTest or Mini-BESTest section. ANOVA showed a significant difference depending on gender in sections I, IV and VI. Values at section I (Biomechanical Constraints) were F(2. 27) = 3.22, *p* = 0.06. The mean differences were 1.9 (*p* < 0.03) obtained by rater 1 and 1.9 (*p* < 0.02) by rater 2 (Table 2). This difference represents 14% of the maximal score of section I.

In section IV (Postural Responses), the value was F(2. 27) = 4.48, *p* = 0.02 for both raters (Scheffé post-hoc for both raters was *p* < 0.01). This difference represents 10% of the maximal score of the Postural Responses section. Also in section VI (Stability of Gait), ANOVA showed an F(2. 27) = 3.47, *p* = 0.05 but only for scores registered by the second rater (Scheffé post-hoc for rater 1 p < 0.01, rater 2 *p* < 0.07).

For Mini-BESTest, scores obtained by women were also lower than those reached by men in all sections and for both raters (Table 2).

### Internal consistency reliability

BESTest and Mini-BESTest showed a good internal consistency, with Cronbach alpha values between 0.79 and 0.98 (Table 3).

**Table 3:** Inter-rater concordance in each BESTest section and for each Mini-BESTest items

	ICC Unique M (95%CI)^a^	ICC Average M (95%CI)^b^	Alfa Cronbach	SEM^c^	MDC^d^
*BESTest*					
Section I. Biomechanical constraints	0.88 (0.76–0.94)	0.94 (0.86–0.97)	0.93	0,61	1,68
Section II. Stability limits/verticality	0.81 (0,64-0,90)	0,79 (0,78-0,95))	0.90	0,85	2,35
Section III. Anticipatory postural adjustments	0.96 (0.92–0.98)	0.98 (0.96–0.99)	0.98	0,26	0,71
Section IV. Postural responses	0.73 (0.51–0.82)	0.84 (0.67–0.93)	0.84	0,73	2,02
Section V. Sensory orientation	0.92 (0.85–0.96)	0.96 (0.92–0.98)	0.96	0,27	0,75
Section VI. Stability in gait	0.89 (0.78–0.94)	0.94 (0.87–0.97)	0.94	0,45	1,23
Total Score	0.93 (0.86–0.97)	0.97 (0.93–0.98)	0.97	1,18	3,27
*Mini-BESTest*					
Anticipatory	0.89 (0.78–0.95)	0.94 (0.88–0.97)	0.94	0,29	0,79
Reactive postural control	0.67 (0.41–0.83)	0.80 (0.58–0.90)	0.80	0,43	1,19
Sensory orientation	0.72 (0.50–0.86)	0.84 (0.66–0.92)	0.84	0,39	1,07
Dynamic gait	0.78 (0.59–0.89)	0.88 (0.74–0.94)	0.88	0,35	0,97
Total score	0.65 (0.38–0.82)	0.79 (0.55–0.90)	0.79	0,74	2,04

^a^Intraclass Correlation Coeficient Unique Measurement (95% Confidence Interval)

^b^Intraclass Correlation Coeficient Average Measurement (95% Confidence Interval)

^c^Standard Error of Measurement

^d^Minimum Detectable Change

### Inter-rater reliability

**Fig. 1:**
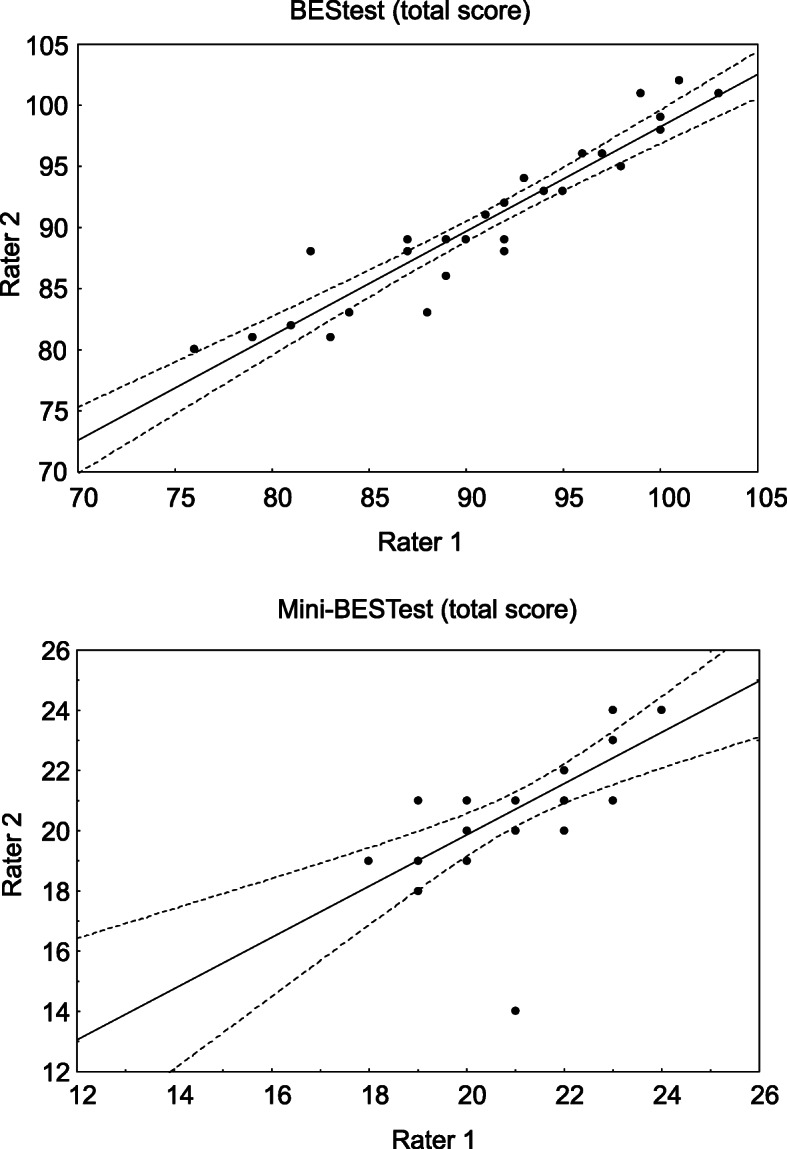
The BESTest total score inter-rater agreement

**Fig. 2:**
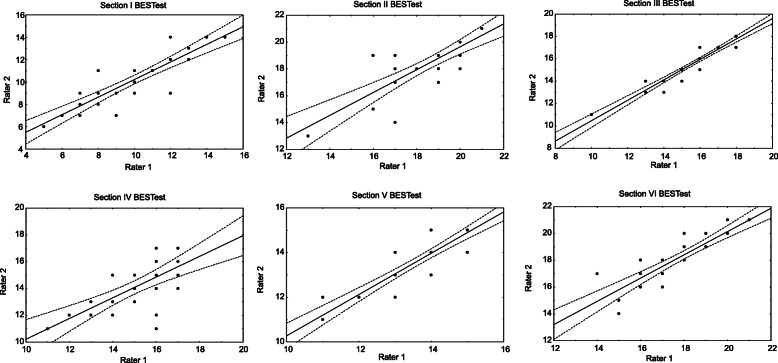
Inter-rater reliability in the BESTest sections

**Fig. 3:**
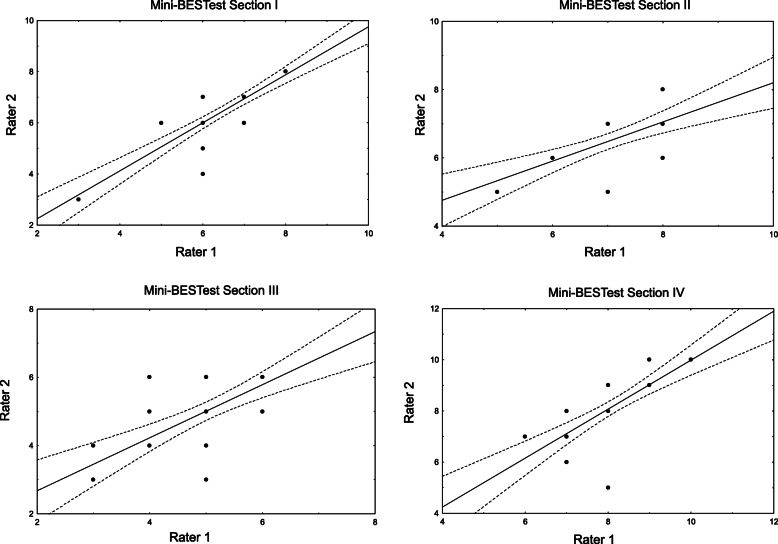
Inter-rater reliability in the Mini-BESTest sections

The BESTest total score showed excellent inter-rater agreement (ICC = 0.97, 95% CI, 0.93–0.98, Table 4, Fig. 1). Also excellent was inter-rater reproducibility for the total score of Mini-BESTest (ICC = 0.79, 95% CI 0.38–0.82, Table 4, Fig. 1). All sections of the BESTest and the Mini-BESTest similarly showed excellent inter-rater reliability (Table 3, Figs. 2, 3). The highest ICC average was for Anticipatory postural adjustments (ICC = 0.98, 95% CI 0.96–0.99 for BESTest and ICC = 0.94, 95% CI 0.88–0.97 for Mini-BESTest, Table 3).

**Table 4:** Correlation between sections of BESTest and Mini-BESTest

	Women (Pearson’s r)	Men (Pearson’s r)	Total (Pearson’s r)
Anticipatory	0.93^*^	0.87^*^	0.90^*^
Postural responses	0.44^†^	0.76^†^	0.59^*^
Sensory orientation	0.90^*^	0.98^*^	0.94^*^
Dynamic gait	0.88^*^	0.94^*^	0.92^*^
Total score	0.51^†^	0.56^†^	0.65^*^

*p < 0.001

†p < 0.05

### Validity

There was a strong positive and statistically significant correlation between the total scores of BESTest and Mini-BESTest (r = 0.65; *p* < 0.001, Table 4). Correlations between corresponding sections of both systems were strongly positive and statistically significant (Table 4).

Discriminating by gender, all sections correlated strongly and obtained statistical significance. Higher correlation was observed in the sensory orientation section, being higher in men (r = 0.98 and 0.90 respectively; p < 0.001, Table 4). There was a moderate correlation in women’s postural responses (r = 0.44, *p* < 0.05, Table 4).

BESTest had a moderate association with BBS (r = 0.43, p 0.02, Table 5) and a strong association with FES-I with statistical significance(r = 0.90). Mini-BESTest had a fair correlation with BBS and FES-I. There was no correlation between Mini-BESTest and BBS in the case of women (Table 5).

**Table 5:** Correlations between BESTest and MiniBest with BBS and FES-I

Scale	Women Pearson’s r (p value)	Men Pearson’s r (p value)	Total Pearson’s r (p value)
BESTest			
FES-I	0.21 (0.44)	0.48 (0.14)	0.90 (0.65)
BBS	0.44 (0.10)	−0.01 (0.96)	0.43 (0.02)^*^
MINIBest			
FES-I	−0.14 (0.60)	0.22 (0.51)	−0.18 (0.37)
BBS	0.003 (0.90)	−0.18 (0.53)	0.18 (0.35)

^*^Significant correlation (p < 0.05)

## Discussion

This study aimed to verify psychometric properties of BESTest and Mini-BESTest once translated to Spanish in a community-based sample of older people without related balance problems and secondarily to study sex-gender differences. Validation was done by comparing to BBS and FES-I.

Our results showed that the Spanish translated version of BESTest and Mini-BESTest are reliable and valid measures of balance performance for community-dwelling elderly people not prone to falling, and that scores of BESTest and Mini-BESTest obtained by women were lower than the ones obtained by men. Data obtained in the BESTest showed similar results for the central tendency indicators than those obtained in previous studies performed by Anson and O’Hoski [5, 7]. Both balance measurement systems showed a good internal consistency for items in respect of the total score and for each of the six sections of BESTest and the four of the Mini-BESTest, also demonstrating that the items measured the same underlying attribute. O’Hosky et al. [5], showed that the BESTest, mini-BESTest and briefBESTest present the construct validity for assessing balance in adults aged over 50 years. We also found a greater degree of agreement between raters for the BESTest validity, indicated by excellent inter-rater reliability for total score and for test subsections. Values for ICC in all sections, except Postural responses, were similar to those obtained by Padgett or Wang-Hsu [23, 25]. Reliability in Mini-BESTest indicated excellent inter-rater concordance data, although with lower scores in some sections than those registered in people with chronic stroke or Parkinson’s disease [11, 22, 26]. Positive correlations between BESTest and Mini-BESTest were found, as obtained by other studies with the same scales developed in other languages [16, 18].

Scores of BESTest and Mini-BESTest obtained by women were lower than men. There is strong evidence that physical activity reduces the risk of falling [27, 28]. Some papers have also pointed out that physical activity is more intense and frequent in elderly men, while activities of daily living are usually more demanding for women [29]. Unfortunately, in our study we have not recorded a validated score for physical activity, but the participants who were men reported practicing more physical activity than women. This is the only factor that could explain our results. Comorbidity, medication or socioeconomic differences, other important-factors mentioned in the literature as responsible for poor balance [29–31] should not be taken into account in our present work because we selected for healthy subjects, unhealthy were excluded, and they were mostly couples with a similar socioeconomic status. Nevertheless, despite the oral report of physical activity, due to the absence of a systematic collection of this information through self-reports or administered scales, we consider that one should take with caution the interpretation that the differences in balance score between genders is due to differences in levels of physical activity. Those differences observed in all the test when disaggregating data by sex require further research.

At the criterion validity and correlating with BBS, a significant correlation was found with BESTest and a fair one with Mini-BESTest, obtaining an inverse correlation in men, as in other research [5]. It could be asked if, in relation to the BESTest and BBS, this inverse correlation is only in any person with good balance or especially in men because, as some studies point out, they have better balance. Those results suggest that other measurement systems should be included to identify in a more accurate and sensitive way individuals with functional limitations beyond BBS [10, 11]. Fear of falling measured by FES-I showed a strong association with BESTest, although this was not statistically significant. Regarding the Mini-BEST, in the total sample a negative correlation was observed with FES-I, so the risk of falling decreased when dynamic scores in balance increased, as expected [13, 32]. BESTest correlated positively and strongly with Mini-BESTest, but this correlation was inverse. It could be that aspects that the Mini-BESTest fails to evaluate, that are included in the BESTest, make this assessment less accurate in people who are afraid of falling. This situation is particularly important in the group of women, since in men the correlation does not have a positive sign. In general, we have observed that the correlation between the scales used in this study is greater the worse the balance is. To detect which aspects of balance can be improved in the healthy ageing population, it would be the BESTest that detects more precisely these difficulties. This is especially true in the diagnosis and treatment of balance problems considering the differences observed between women and men. We want to highlight these results, which lead us to ask ourselves if better balance in independent elderly people who have no history of a recent fall is linked to gender. There is no consensus in the literature: some studies did not find them, while others observed indeed lower levels of postural stability and balance for men [29–31].

It would be also crucial to identify those factors that decrease or increase risk of falling and that contribute to maintaining functional independence.

### Study limitations and directions for future research

The not-fully-random sampling that was adopted for practical reasons limits the strength with which these results can be used to infer properties of the general population.

This study was designed to assess the psychometric properties of BESTest and Mini-BESTest in community-dwelling elderly people in Spanish. However, given the differences obtained in the correlations between BESTest and MiniBESTest and FES-I or BBS when disaggregating data by gender, it should be studied if, at similar ages, the same level of physical activity in that population implies a similar quality of balance. This would require a larger sample size than the one in the present study.

We strongly recommend cultural adaptation to other Spanish-speaking countries, especially in Central and South America.

## Conclusions

Spanish versions of BESTest and Mini-BESTest are balance systems comprehensible for new raters. The BESTest and Mini-BESTest are valid and reliable tools to provide information on which particular balance systems were the underlying cause of balance impairments in community dwelling older adults. Our results suggest that elderly women showed a worse quality of balance and a greater perception of their risk of falling than men.

## Data Availability

The datasets analysed during the current study are available from the corresponding author on reasonable request.

## Abbreviations

BESTest: Balance Evaluation Systems Test
BBS: Berg Balance Scale
CEICA: Comité Ético de Investigación de Aragón-Spain
CI: Confidence Interval
FES-I: Falls Efficacy Scale International
ICC: Intraclass Correlation Coefficient
MDC: Minimum Detectable Change
